# A Critical Role of Bacterioferritin in *Salmonella pullorum*-Induced IFN-β Expression in DF-1 Cells

**DOI:** 10.3389/fmicb.2016.00020

**Published:** 2016-02-03

**Authors:** Zhichao Xu, Yao Qin, Yongqiang Wang, Xiaoqi Li, Hong Cao, Shijun J. Zheng

**Affiliations:** ^1^State Key Laboratory of Agrobiotechnology, China Agricultural UniversityBeijing, China; ^2^Key Laboratory of Animal Epidemiology and Zoonosis, Ministry of Agriculture, China Agricultural UniversityBeijing, China; ^3^College of Veterinary Medicine, China Agricultural UniversityBeijing, China

**Keywords:** *Salmonella pullorum*, bacterioferritin, interferon, IFN-β

## Abstract

*Salmonella enterica* serovar Pullorum (*S. pullorum*) causes pullorum disease in poultry and results in great economic losses to the poultry industry. Although an eradication program has been successfully performed in some countries, it remains a major threat to countries with poor poultry disease surveillance. Currently there are no effective control measures for pullorum disease except eradication. In particular, the pathogenesis of *S. pullorum* infection is still largely unknown. Here we identified bacterioferritin (Bfr) as a major antigen of *S. pullorum* to elicit a humoral immune response. Furthermore, we demonstrate that Bfr induces activation of IFN-β promoter and mRNA expression in DF-1 cells, and that the amino acids 1–50 form a critical domain involved in IFN-β expression. Moreover, we found that the p38 MAPK signaling pathway was essential for Bfr-induced IFN-β expression. Importantly, *S. pullorum*-induced IFN-β expression was totally abolished by deficiency of Bfr in the bacteria, indicating that Bfr plays a critical role in *S. pullorum* induced IFN-β expression in DF-1 cells. Our findings provide new insights into the molecular mechanisms of the host response to *S. pullorum* infection.

## Introduction

Pullorum disease, an acute systemic disease commonly seen in young birds, is caused by *Salmonella enterica* serovar Pullorum. The clinical signs of pullorum disease are characterized by anorexia, diarrhea, dehydration, weakness and high mortality in young chicks, but this disease usually shows a persistent infection and causes decreased egg production and diarrhea in adult fowls (Shivaprasad, 2000). Pullorum disease is basically controlled in Europe and North America, but it still occurs in many countries such as Brazil, Argentina, India, and China, leading to severe economic losses (Barrow and Freitas, 2011; Barrow et al., 2012). *Salmonella* spp. belongs to the Enterobacteriaceae family. *Salmonella* is a Gram-negative and facultative intracellular pathogen which, depending on the serotype and host, can cause diseases ranging from gastroenteritis to typhoid fever (Marcus et al., 2000). *S. pullorum*, currently belonging to biovars of serovar Gallinarum within serogroup D, has identical somatic antigens (O1, O9, O12) and no flagella due to mutations in flagellar genes while its pathogenicity is restricted only to avian species (Barrow and Freitas, 2011). The relatively high rate of accumulation of mutations in the genome of *S. pullorum* suggests a rapid rate of evolution associated with the host adaptation, particularly in the development of *S. pullorum* (Barrow and Freitas, 2011). During *S. pullorum* infection, the interaction of this pathogen with the immune system occurs in three main phases, including invasion via the gastrointestinal tract, establishment of systemic infection and induction of cytokine expression (Chappell et al., 2009).

High titers of anti-*Salmonella* IgY were produced by birds infected with *S. pullorum* from 5 weeks onwards and *S. pullorum* was detected in splenic macrophages from 3 days to 10 weeks postinfection (Wigley et al., 2001). It was found that approximate 1 to 2% of macrophages contained fluorescent *Salmonella* bacteria in all birds examined, and dropped to less than 1% at 5 weeks postinfection and even further at 10 weeks to less than 0.5% of cells infected (Wigley et al., 2001), indicating that macrophage plays a critical role in clearance of *S. pullorum*. An antigen-specific T-cell response to *S. pullorum* was found in birds at 5 and 9 weeks postinfection, but dropped to negligible levels at 17 weeks postinfection (Wigley et al., 2005). The numbers of *S. pullorum* bacteria recovered from the spleen, liver, the reproductive tracts and developing eggs increased following the fall in T-cell proliferation activity at 18 weeks postinfection, while T-cells proliferation began to increase at 22 weeks postinfection (Wigley et al., 2005). In contrast to T-cell response, antibody response did not decline (Wigley et al., 2005). Like other pathogens, *Salmonella* infection stimulates cytokine production. The induction of cytokines such as IL-1β, IL-8, IL-12, IL-17, IL-18, TNF-α, and IFN-γ following *Salmonella* infection of chickens have been previously reported (Withanage et al., 2004; Berndt et al., 2007; Crhanova et al., 2011). One of the most remarkable features of *Salmonella* infection is that IFN-β was induced in fibroblasts and macrophages (Hess et al., 1989; Robinson et al., 2012). The role of IFN-β in the response to bacterial infection is variable, and it contributes to a variety of beneficial and detrimental immune functions (Monroe et al., 2010).

The iron that is acquired by the pathogenic bacterium is used for numerous biochemical activities and any surplus iron that is available is stored within the bacterial cell in the form of Bfr (Ratledge, 2007). Bfr belongs to an outer membrane protein in *S. hadar* as examined by a proteomic approach (Snoussi et al., 2012). Bfr is a major iron storage protein and protects against hydrogen peroxide toxicity, and the haeme-containing Bfr was found exclusively in bacteria (Velayudhan et al., 2007).

Currently it is known that Bfr is a T-cell antigen that induced a strong IFN-γ production and the proliferation of lymphocytes (Denoel et al., 1997; Al-Mariri et al., 2002; Lee et al., 2006). In addition, Bfr induced humoral immune response in mice immunized with DNA vaccine encoding the Bfr or recombinant Bfr proteins (Al-Mariri et al., 2001a,b. The antibodies against Bfr were detected from Crohn’s disease, and 53% of Crohn’s disease patients were positive, indicating that Bfr was a specific protein antigen of *Mycobacterium paratuberculosis* (Walmsley et al., 1996). However, little is known about the role of Bfr in innate immune responses. DF-1, an immortal chicken embryo fibroblast cell line, is commonly used for the research of *Salmonella* (Li et al., 2006; Szmolka et al., 2015) and type I interferon (Li et al., 2013). To gain a better understanding about the role of Bfr in innate immune responses, we set out to determine if Bfr induces humoral immune response in chickens and induces type I IFN expression in *S. pullorum* infected DF-1 cells.

In this study, we demonstrate that Bfr is a major antigen of *S. pullorum*, and Bfr induced IFN-β mRNA expression in DF-1 cells. In addition, we show that the amino acids 1–50 form a critical domain involved in activation of the IFN-β promoter. Furthermore, we found that Bfr induced IFN-β expression likely via the p38 mitogen-activated protein (MAP) Kinase signaling transduction pathway. Importantly, we found that *S. pullorum-* induced IFN-β expression was totally abolished by deficiency of Bfr in the bacteria, indicating that Bfr plays a critical role in *S. pullorum*-induced IFN-β expression in DF-1 cells.

## Materials and Methods

### Bacteria and Cells

*Salmonella pullorum* strain 533 was obtained from China Institute of Veterinary Drug Control (Beijing, China). *Escherichia coli* DH5α and *E. coli* BL21 (DE3) strains were obtained from TransGen Biotech (Beijing, China). Bacteria were grown in LB medium. DF-1 cells were obtained from ATCC (USA), and were cultured in DMEM supplemented with 10% fetal bovine serum (FBS) in 5% CO_2_ incubator.

### Reagents

Protein A/G beads were purchased from GE Healthcare (USA). Protease inhibitor cocktail C was purchased from Yataihengxin Company (Beijing, China). The restriction enzymes *BamH* I, *Xho* I, and *Sph* I were purchased from TaKaRa (Dalian, China). The Mops, FeCl_2_ and H_2_O_2_ were purchased from Solarbio Company (Beijing, China). The Large Amount Without Endo-Toxin Plasmid Preparation Kits and the monoclonal antibody against GAPDH were purchased from CWBio (Beijing, China). DMEM medium was purchased from Hyclone (USA). The jetPRIME reagent was purchased from Polyplus-transfection (France). The serum against *S. pullorum* was collected from chickens with pullorum disease and the control serum from SPF chickens from Beijing Agricultural University Animal Technology Company (Beijing, China). Monoclonal antibody against His-tag fusion protein was purchased from Abmart (Shanghai, China). Anti-GFP monoclonal antibody, anti-p38 monoclonal antibody, and anti-p-p38 monoclonal antibody were purchased from Santa Cruz Biotechnology (USA). Monoclonal antibody against Bfr (Clone ID: EU-0218), pGL3-chIFN-α-luc and pGL3-chIFN-β-luc were obtained from CAEU Biological Company (Beijing, China). HRP-conjugated goat-anti mouse polyclonal antibodies and HRP-conjugated goat-anti rabbit polyclonal antibodies were purchased from DingGuoShengWu Company (Beijing, China). Rabbit-anti chicken polyclonal antibodies were purchased from Bioss (Beijing, China). pET28a (+) vector was obtained from Novagen (USA). pEGFP-N1 vector was purchased from Clontech (USA). p38 (mitogen-activated protein kinase, MAPK) inhibitor SB203580 and JNK inhibitor SP600125 were purchased from Enzo Life Sciences (USA). Red homologous recombination using the plasmids pKD46, pKD3, and pCP20 were kindly provided by Professor Guo-Qiang Zhu (Yangzhou University, China).

### Pull-Down Assay

*Salmonella pullorum* was grown in LB medium. The bacterial culture were centrifuged at 6000 × *g* for 5 min, and the pellet was resuspended and lysed in pH 7.4 PBS buffer by ultrasonic treatment. Then 50 μL of 25% protein A/G beads were preincubated with 60 μg rabbit-anti chicken polyclonal antibodies and 200 μL pullorum-positive serum or negative serum as controls for 8 h at 4°C. The mixture was washed three times with pH7.4 PBS by centrifugation at 825 × *g* for 3 min at 4°C and the supernatant was removed after the last wash. The rabbit-anti chicken polyclonal antibody-conjugated beads with anti-*S. pullorum* antibodies mixed with the cell extract from *S. pullorum* and incubated at 4°C for 8 h. The mixture was washed as above described. The immunoprecipitates were suspended with 40 μL 1x SDS-PAGE loading buffer and boiled for 10 min before resolved on 12% SDS-PAGE gel. Then the gel was stained with Coomassie blue dye for analysis of specific bands.

### Mass Spectrometric Identification of Proteins

After separation of proteins on SDS-PAGE gel, the interesting bands were cut out and subjected to liquid chromatography-mass spectrometry. Briefly, the interesting peptides extracted from gel were dissolved in 0.1% formic acid, and then separated by a Nano-LC system (Micro-Tech Scientific, Vista, CA, USA) equipped with a C_18_ reverse phase column. The peptides were eluted using a 120 min gradients from 0 to 50% acetonitrile in 0.1% formic acid at a constant flow rate of 400 nL/min. Mass spectra were recorded on a 7-T Fourier transform ion-cyclotron resonance (FTICR) mass spectrometer, Apex-Qe (Bruker Daltonics, Bremen, Germany). Data were acquired in data-dependent mode using ApexControl 1.0 software (Bruker Daltonics, Bremen, Germany). Three strongest peaks of each MS acquisition were selected for the following MS/MS analysis. The MS/MS spectra were processed by DataAnalysis 3.4 (Bruker Daltonics, Bremen, Germany) with S/N ≥ 4.0, and automatically searched against IPI.RAT database (version 3.41) using the Mascot 2.1.0 (Matrix Science, London, U.K.). The NCBI database was used in the search.

### Construction of Plasmids

The *bfr* gene was amplified from *S. pullorum* genomic DNA by PCR using the specific primers containing *BamH* I in sense and *Xho* I in antisense (sense: 5′-CGCGGATCCATGAAAGGTGATGTTAAA-3′; antisense: 5′-CCG CTCGAGATCGGTAACCTTAATTTG-3′) that were designed with reference to the published sequence (GenBank, gene ID: 661554730), and the PCR products were cloned into the pET28a (+). The resulting plasmid was named pET28a-*bfr*. The *bfr* gene was then subcloned into pEGFP-N1 vector using primers with *Xho* I in sense and *BamH* I in antisense (sense: 5′-CCGCTCGAGATGAAAGGTGATGTTAAA-3′; antisense: 5′-CGCGGATCCCGATCGGTAACCTTAATT-3′) and this plasmid was named pEGFP-*bfr*. Different truncated *bfr* segments were subcloned into pEGFP-N1 vectors and were named Bfr (1–50aa), Bfr (51–158aa), Bfr (101–158aa) accordingly. The Bfr (1–50aa) plasmid was constructed using the same sense primer as pEGFP-*bfr* plasmid and the antisense primer is 5′-GGATCCCGATCAATGGATTCATGGTAC-3′ containing *BamH* I restriction site. The Bfr (51–158aa) and the Bfr (101–158aa) plasmids were constructed using the same antisense primer as pEGFP-*bfr* plasmid. For Bfr (51–158aa) plasmid, the sense primer is 5′-CCGCTCGAGGAGATGAAACACGCCGATA-3′. For Bfr (101–158aa) plasmid, the sense primer is 5′-CTCGAGCTACGTGAGGCAATAGCC-3′. All the sense primers contained *Xho* I restriction site. All the primers were synthesized by Sangon Company (Shanghai, China), and all the constructs were confirmed with sequencing analysis by Huada Company (Beijing, China).

### Iron Uptake Assays

Iron uptake by rBfr was examined using SpectraMax M5 according to the method described by Timoteo et al. (2012). Briefly, reaction of 0.5 μM rBfr with 12 μM Fe^2+^ ions and 72 μM H_2_O_2_ in 0.2 M Mops buffer (pH 7) and 0.2 M NaCl, and BSA was used as control. The iron storage capacity was determined by plotting the OD optical density at 200–400 nm in Spectra Max M5.

### Transfection and Reporter Gene Assays

DF-1 cells (1 × 10^5^) were seeded on 24-well plates and cultured overnight before transfection with pEGFP-*bfr* or pEGFP-N1, together with pGL3-chIFN-β-luc (or pGL3-chIFN-α-luc) and pRL-TK using jetPRIME reagents (Polyplus-transfection). Twenty four hours after transfection, cell extracts were harvested, and the luciferase activities were examined with a dual-specific luciferase assay kit (Promega). Firefly luciferase activities were normalized based on Renilla luciferase activities. All reporter gene assays were repeated at least three times. Data are represented as mean ± SD.

### RNA Isolation and RT-PCR Analysis

Total RNA was prepared from DF-1 cells using a RNeasy kit (Aidlab, China) per the manufacturer’ instruction, and was treated with DNase I. Two μg of total RNA was used for cDNA synthesis by reverse transcription using RT-PCR kit (TaKaRa). The specific primers for chicken IFN-α1 (sense: 5′-CCAGCACCTCGAGCAAT-3′; antisense: 5′-GGCGCTGTAATCGTTGTCT-3′), IFN-β (sense: 5′-GCCTCCAGCTCCTTCAGAATACG-3′; antisense: 5′-CTGGATCTGGTTGAGGAGGCTGT-3′), and glyceraldehyde-3-phosphate dehy drogenase (GAPDH; sense: 5′-TGCCCATCACAGCCACACAGAAG-3′; antisense: 5′-ACTTTCCCCACAGCCTTAGCAG-3′) were designed with reference to previous publications (Li et al., 2007; Abdul-Careem et al., 2008; Liu et al., 2010) and synthesized by Sangon Company (Shanghai, China). The real-time PCR assay was carried out with a Light Cycler 480 (Roche, USA). The PCR was performed in a 20-μL volume containing 1 μL of cDNA, 10 μL of 2 × SYBR green Premix *Ex Taq* (TaKaRa), and a 0.4 μM of each gene-specific primer. The thermal cycling parameters were referred to the previous study (Li et al., 2013), and they were as follows: 94°C for 2 min; 45 cycles of 94°C for 20 s, 55°C for 20 s, and 72°C for 20 s; and 1 cycle of 95°C for 30 s, 60°C for 30 s, and 95°C for 30 s. The final step was to obtain a melt curve for the PCR products to determine the specificity of the amplification. All sample reactions were carried out in triplicate on the same plate, and the GAPDH gene was utilized as the reference gene. Expression levels of genes were calculated relative to the expression of the GAPDH gene and expressed as fold increase or decrease relative to the control samples.

### Inhibition of Signal Transduction Pathways

DF-1 cells (4 × 10^5^) were seeded on a 6-well plates and cultured for 24 h before treatment with p38 inhibitor SB203580 (20 μM), JNK inhibitor SP600125 (20 μM), and dimethyl sulfoxide (DMSO) as control for 1 h, and then transfected with pEGFP-*bfr* or pEGFP-N1 as controls using jetPRIME reagents. Twenty four hours after transfection, total RNA was extracted and used for cDNA synthesis. Real-time PCR was performed to examine the expressions of chicken IFN-β and GAPDH at an mRNA level as above described.

### Western Blot Analysis

The pET28a-*bfr* recombinant or empty vectors were transformed into *E. coli* BL21 (DE3), and Bfr-his recombinant proteins were expressed by 1 mM IPTG induction at 37°C for 6 h. One mL bacterial cells were centrifuged at 6000 × *g* for 5 min and the pellets were resuspended with 120 μL 1x SDS-PAGE loading buffer and boiled for 10 min before fractionated by electrophoresis on 12% SDS-PAGE gels, and the resolved proteins were transferred onto PVDF membranes (Millipore, USA). After blocking with 5% skim milk, the membranes were probed with primary antibodies [anti-His-tag (1:10000), pullorum-positive chicken serum (1:500), or pullorum-negative SPF chicken serum (1:500)], followed by incubation with HRP-conjugated goat anti-mouse IgG (1:20000; DingGuoShengWu, China) or HRP-conjugated rabbit anti-chicken IgY (1:5000) secondary antibodies (Bioss, China). The blots were visualized using the ECL reagent according to the manufacturer’s instructions (CWBio, China). For GFP-Bfr expression analysis, cells were transfected with pEGFP-*bfr* or pEGFP-N1, cells lysates were prepared and examined with anti-GFP antibodies. For signal transduction pathways analysis, cells were transfected with pEGFP-*bfr* or pEGFP-N1. The whole cell extracts were lysed in lysis buffer (150 mM NaCl, 5 mM EDTA, 50 mM Tris⋅Cl, 10% glycerin, 1% Triton X-100) containing with 1% protease inhibitor cocktail C and 1% 20 mM phosphatase inhibitors (NaF) and examined with Western Blot using anti-p-p38, anti-p38, anti-GFP, and anti-GAPDH antibodies.

### Generation of the Bfr-Deficient *S. pullorum*

The Bfr-deficient strain was constructed by λ-Red-mediated recombination system, according to the method described by Datsenko and Wanner (2000). Briefly, the specific primers (ΔBfr1: 5′-ATGAAAGGTGATGTTAAAATCATAAATTATCTCAAT AAACTATTGGGAAATGTTAGGCTGGAGCTGCTTCG-3′; ΔBfr2: 5′-TTAATGGTAACCTTAATTTGTGATTGCAGATAATTTTGCATACCAAGTTCATATGA ATATCCTCCTTAG-3′), including 50-bp homology extension from the 5′ and 3′ of the *bfr* gene, were designed to amplify the chloramphenicol cassette from the template plasmid pKD3 by PCR. The PCR products were purified and electroporated into *S. pullorum* containing the pKD46 plasmid. Recombinant bacteria *S. pullorum* Bfr::cat was screened and selected on both Cm and Amp resistance LB agar plates. Gene deletion was confirmed by PCR using the specific primers (Bfr1: 5′-ATGAAAGGTGATGTTAAA-3′; Bfr2: 5′-ATCGGTAACCTTAATTTG-3′). Then the Cm cassette gene of *S. pullorum* Bfr::cat was excised via introducing the Flp recombinase-expressing vector pCP20 by electroporation. The Bfr-deficiency in parental strain was confirmed by PCR, DNA sequencing and Western Blot.

To generate the ΔBfr-complemented strain, the Bfr open-reading frame was amplified by PCR using *S. pullorum* genomic DNA with the specific primers containing *BamH* I and *Sph* I (PBR-Bfr1: 5′-CGCGGATCCATGAAAGGTGATGTTAAA-3′; PBR-Bfr2: 5′-ACATGCATGCTTAATCGGTAACCTTAATTTG-3′). The expected 477 kb PCR product of *bfr* gene was confirmed by DNA sequencing, and further cloned into the plasmid pBR322. The constructed recombinant plasmid was electroporated into the Bfr-deficient bacteria to obtain the ΔBfr-complemented strain. The restoration of Bfr in parental strains was confirmed by PCR and Western Blot.

### Infection of DF-1 Cells with Wild Type (WT), Bfr-Deficient and Complemented *S. pullorum*

DF-1 cells were infected with WT, Bfr-deficient (KO) and ΔBfr-complemented (RS) strains at an MOI of 500 or indicated doses. Eight hours after infection, total RNA was extracted from the infected cells and used for cDNA synthesis. Real-time PCR was performed to examine chicken IFN-β expression as above described.

### Statistical Analysis

The significance of the differences between the treatment group and control in the activation of promoters and mRNA expressions (cytokine and transcription factor) was determined by the ANOVA and Mann-Whitney accordingly.

## Results

### Screening for the Major Antigens of *S. pullorum*

**FIGURE 1:**
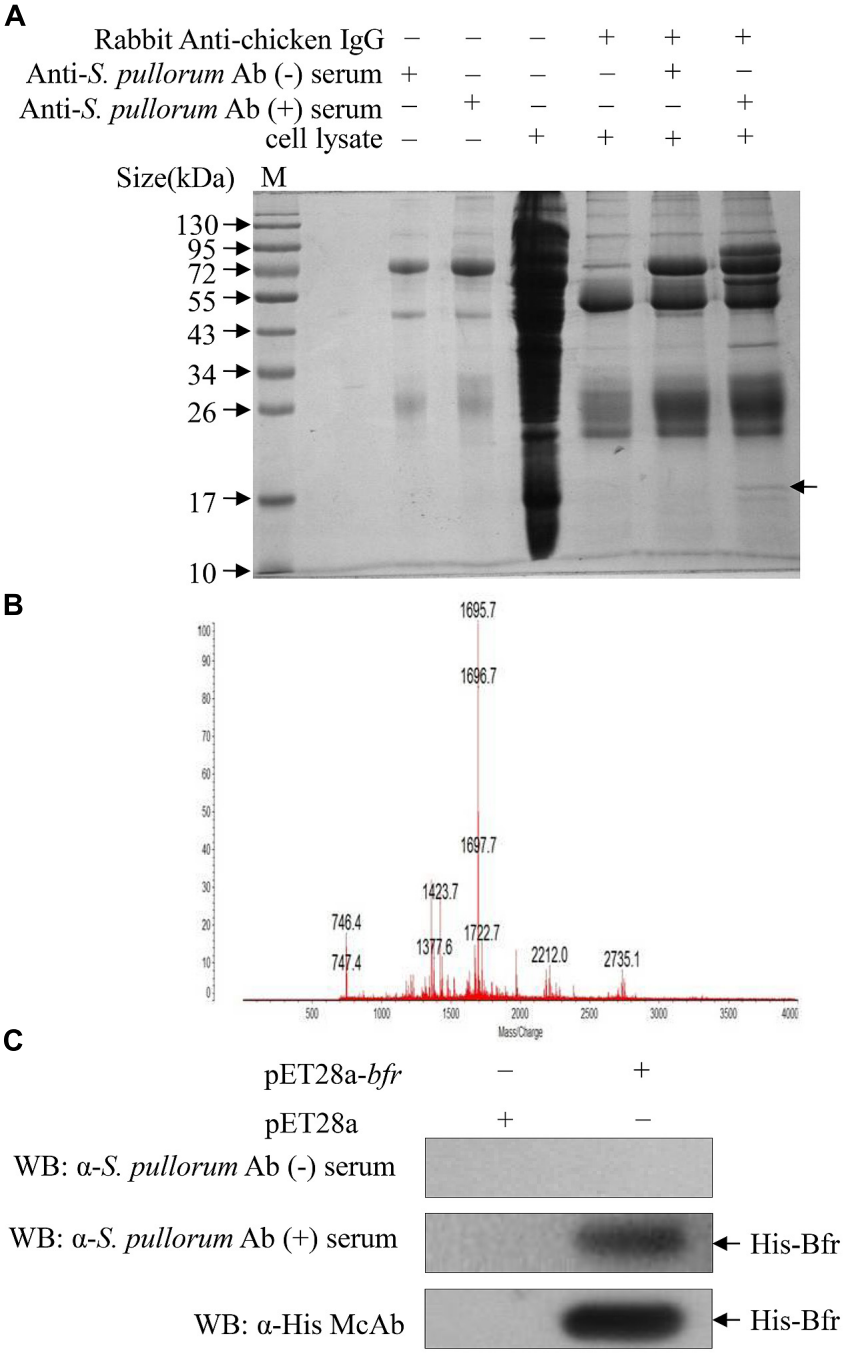
**Identification of major antigens of *Salmonella pullorum* by Pull-down assay. (A)** Pull-down assay was performed to indentify major antigens using extracts of *S. pullorum* with anti-*S. pullorum* Ab positive serum of chickens and negative serum as controls. The pull-down pellets were examined by SDS-PAGE. **(B)** The arrow-pointed band was cut down and subjected to Mass Spectrometry analysis. **(C)** Recognition of recombinant His-bacterioferritin (Bfr) fusion protein by anti-*S. pullorum* Ab positive serum. Expressions of recombinant His-Bfr fusion protein were detected by Western Blot using anti-*S. pullorum* Ab positive serum, negative serum and anti-His McAb as control.

Since *S. pullorum* infection elicits a robust humoral immune response in chickens, we wanted to determine the major antigens of *S. pullorum* responsible for the induction of specific antibodies. We proposed that major antigens of *S. pullorum* could be pulled down by specific IgY in the anti-*S. pullorum* Ab positive serum. To test our hypothesis, we performed a pull-down assay using anti-*S. pullorum* Ab positive serum of chickens and the bacterial cell extract of *S. pullorum* according to the published method (Cho et al., 2013). We found that there was an extra clear protein band in the immunoprecipitates of the mixture of anti-*S. pullorum* Ab-positive serum with bacterial cell lysate as compared to that of controls as demonstrated by SDS-PAGE (**Figure 1A**), indicating that the antigens of *S. pullorum* could be pulled down by anti-*S. pullorum* antibodies. To analyze the amino acid sequence of this major antigen, we cut-down the interesting protein band and performed a mass spectrometry. As a result, the arrow-pointed band in **Figure 1A** is a protein named bacterioferritin (Bfr) based on online information from GenBank (Gene ID: 661554730; **Figure 1B**). Although Bfr is known as a protein antigen of *M. paratuberculosis* (Walmsley et al., 1996), our data indicate that this protein might be an important major antigen of *S. pullorum*.

To determine the antigenicity of Bfr, we cloned *bfr* gene from genomic DNA of *S. pullorum* and expressed Bfr-his recombinant protein using an *E. coli* expression system. We found that the Bfr-his fusion protein could be detected by anti-*S. pullorum* Ab positive serum of chickens but not by negative chicken serum (**Figure 1C**), indicating that Bfr-his protein is of good antigenicity. These results suggest that Bfr may serve as a major antigen of *S. pullorum*.

**FIGURE 2:**
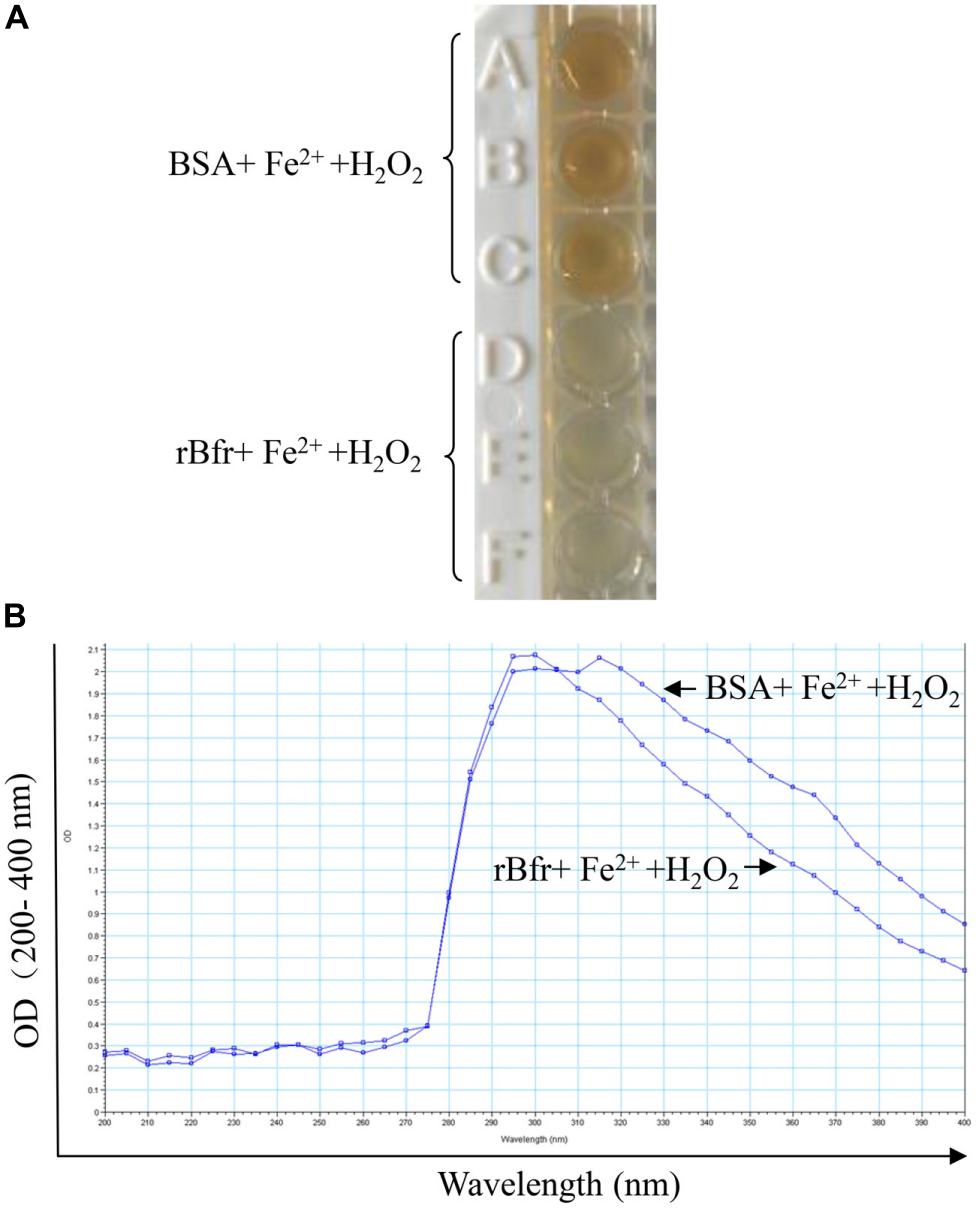
**Bacterioferritin uptakes iron *in vitro*. (A)** Reaction of 0.5 μM rBfr with 12 μM Fe^2+^ ions and 72 μM H_2_O_2_ in 0.2 M Mops buffer (pH 7) and 0.2 M NaCl was carried out, and BSA was used as a control. **(B)** The OD optical density was examined at 200–400 nm in SpectraMax M5.

To determine the basic function of Bfr-his(rBfr) fusion protein, we performed the iron uptake assays with rBfr according to the published method (Timoteo et al., 2012). We found that the color of Fe^3+^ in the BSA control could be clearly observed. In contrast, Fe^3+^ color was markedly reduced with rBfr treatment (**Figures 2A,B**), indicating that Bfr can rapidly uptake free Fe^2+^ for oxidation in the presence of H_2_O_2_ as the oxidant. These data suggest that rBfr is a functional iron storage protein.

### Bfr Induces IFN-β Expression in DF-1 Cells

**FIGURE 3:**
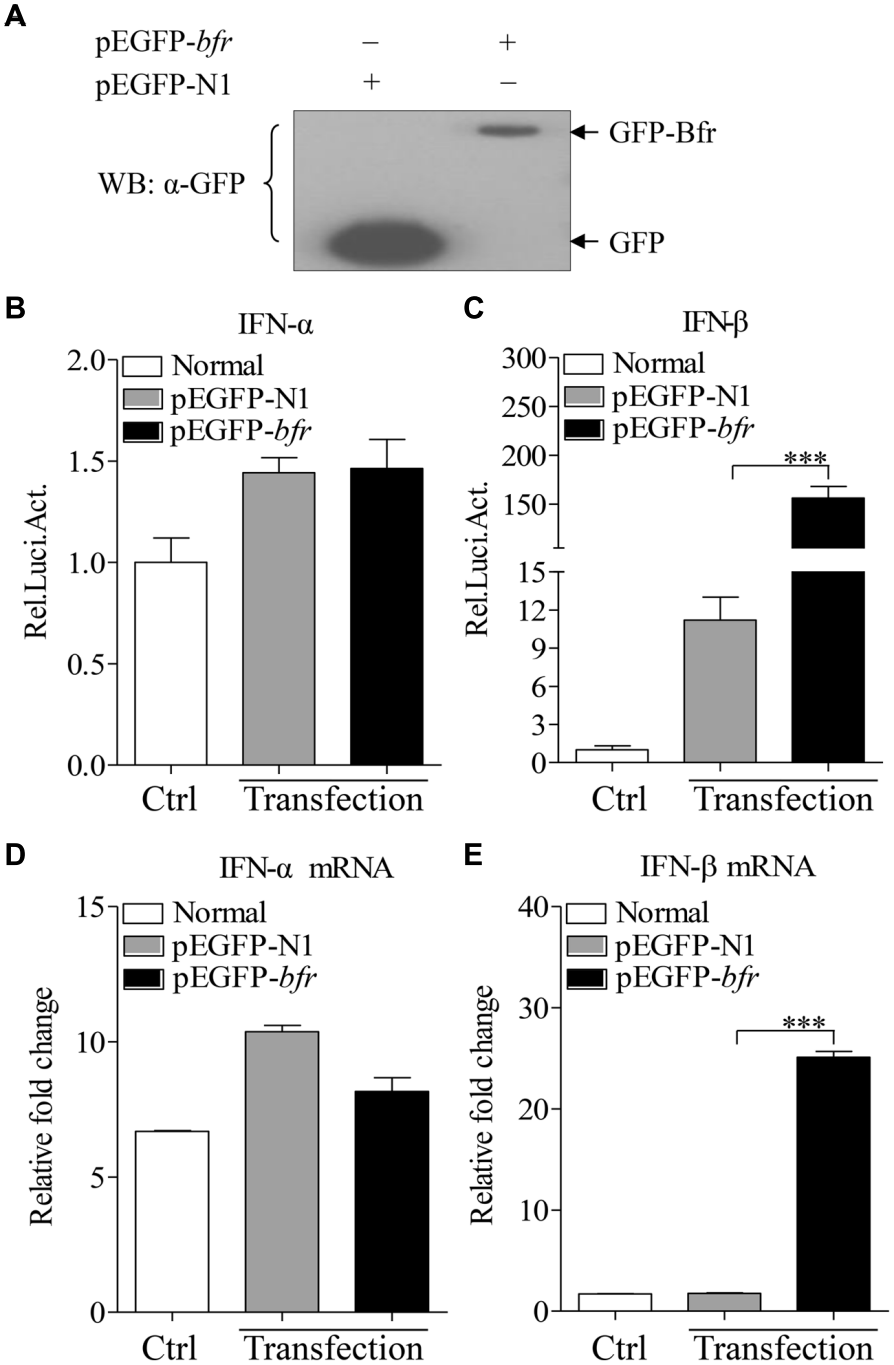
**Expression of Bfr induces activation of IFN-β promoter and enhances mRNA expressions of IFN-β in DF-1 cells. (A)** DF-1 cells were transfected with pEGFP-*bfr* or pEGFP-N1. Twenty four hours after transfection, cell lysates were prepared and examined with Western Blot using anti-GFP monoclonal antibody. **(B,C)** DF-1 cells were transfected with pEGFP-*bfr* or pEGFP-N1 as controls. Twenty-four hours after transfection, cell lysates were prepared and activation of IFN-α and IFN-β promoters were examined with luciferase reporter gene assays. **(D,E)** DF-1 cells were transfected with pEGFP-*bfr* or pEGFP-N1 as controls. Twenty-four hours after transfection, cells were collected and mRNA expressions of IFN-α and IFN-β were examined with qRT-PCR using specific primers. The expression levels of mRNA were calculated in relation to the expression level of GAPDH. Results are representative of three independent experiments. Data are represented as mean ± SD, *n* = 3. ^∗∗∗^ *p* < 0.001.

Bfr is a major iron storage protein in bacteria (Velayudhan et al., 2007). It was reported that iron could affect innate immune response by influencing IFN-γ mediated pathways in macrophages (Nairz et al., 2014). This information prompted us to examine the effect of *S. pullorum* Bfr on the innate immune response in host cells. We made a pEGFP-*bfr* expression construct, and transfected DF-1 cells with pEGFP-*bfr* or pEGFP-N1 as control. As shown in **Figure 3A**, both GFP-Bfr and GFP were expressed well in the transfected cells as demonstrated by Western Blot using anti-GFP monoclonal antibody. Importantly, transfection of DF-1 cells with pEGFP-*bfr* markedly enhanced activation of IFN-β promoter, but not IFN-α, as compared to that of controls (**Figures 3B,C**). Consistent with this observation, the mRNA expressions of IFN-β in pEGFP-*bfr* transfected cells significantly increased as compared to that of pEGFP-N1 transfected controls, but the mRNA expression of IFN-α was unaffected (**Figures 3D,E**). These results suggest that intracellular Bfr induces IFN-β response in host cells.

### Amino Acids 1–50 of Bfr are Responsible for Inducing IFN-β Expression

**FIGURE 4:**
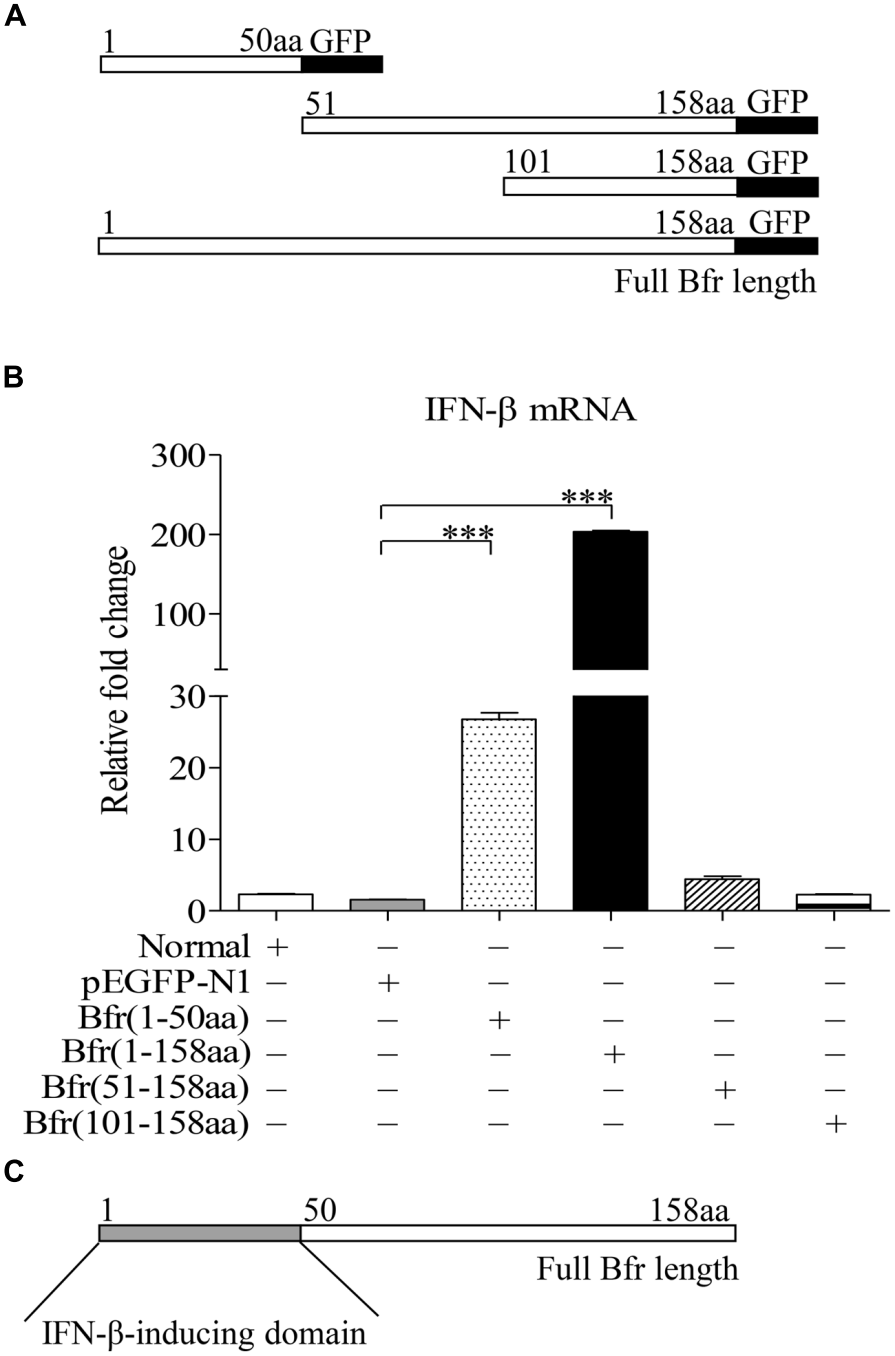
**Bacterioferritin 1–50aa is responsible for the induction of IFN-β expression in DF-1 cells. (A)** Schematic diagrams showing the structure of full-length Bfr and truncated Bfrs. **(B)** DF-1 cells were transfected with indicated amounts of pEGFP-*bfr*, pEGFP-truncated *bfrs* or pEGFP-N1 as controls. Twenty four hours after transfection, cells were collected and mRNA expression of IFN-β was examined with qRT-PCR using specific primers. The expression levels of mRNA were calculated in relation to the expression level of GAPDH. **(C)** Schematic diagrams showing the IFN-β-inducing domain of Bfr. Data are represented as mean ± SD, *n* = 3. ^∗∗∗^ *p* < 0.001.

To determine the domain of Bfr that is responsible for inducing IFN-β expression, we constructed pEGFP-truncated *bfrs* encoding different lengths of Bfr as indicated in **Figure 4A**. We transfected DF-1 cells with the constructs and performed real-time PCR assay with specific primers. As shown in **Figures 4B,C**, transfection of cells with the vectors carrying the full-length and amino acids 1–50 portion of Bfr significantly induced IFN-β expression as compared to that of pEGFP-N1 transfected controls. In contrast, the construct without the gene encoding the amino acids 1–50 portion of Bfr failed to induce IFN-β expression. These results indicate that the amino acids 1–50 of Bfr is a critical domain responsible for inducing IFN-β response.

### Bfr-Activated p38 MAP Kinase is Involved in IFN-β Expression

**FIGURE 5:**
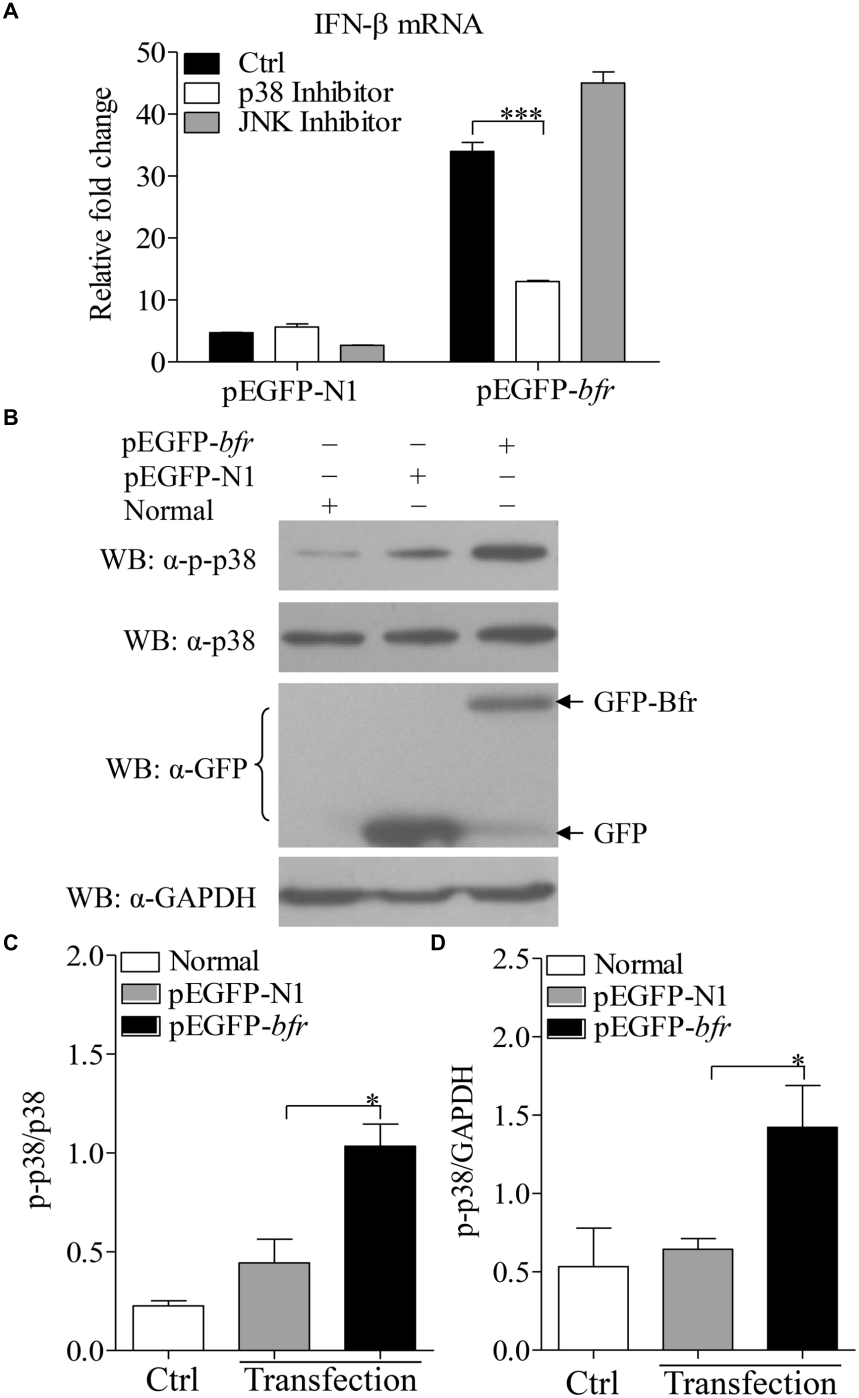
**Bacterioferritin induced IFN-β expression via p38 MAP Kinase signaling pathway. (A)** Effects of p38 and JNK inhibitors on Bfr-induced IFN-β expression. DF-1 cells were transfected with indicated amounts of pEGFP-*bfr* or pEGFP-N1 as controls, and treated with inhibitors of p38, JNK, or DMSO as controls for 1 h. Twenty four hours after transfection, mRNA expression of IFN-β was examined with real-time PCR. **(B–D)** Effects of Bfr on phosphorylation of p38. DF-1 cells were transfected with pEGFP-*bfr* or pEGFP-N1 as controls, Twenty four hours after transfection, cell lysates were prepared and examined with Western Blot for the detection of p-p38, p38, GFP, GFP-Bfr, and GAPDH. The band density of p-p38, p38 and GAPDH in normal, pEGFP-*bfr* or pEGFP-N1 transfected cells in **(B)** was quantitated by densitometry, and the relative levels of p-p38 in **(B)** were calculated as follows: band density of p-p38/band density of p38 **(C)** or GAPDH **(D)**. Results are representative of three independent experiments. Data are represented as mean ± SD, *n* = 3. ^∗∗∗^ *p* < 0.001 and ^∗^ *p* < 0.05.

It was reported that activation of p38 MAP kinase was required for induction of *ifnb* gene expression in response to bacteria in the cytosol (O’Riordan et al., 2002). To dissect the signaling pathways involved in Bfr-induced IFN-β expression, we treated DF-1 cells for 1 h with the inhibitors of the key signaling molecules including p38 MAPK, JNK MAPK or DMSO as controls before pEGFP-*bfr* transfection. Twenty-four hours after transfection, real-time PCR was performed to examine the mRNA levels of IFN-β. As shown in **Figure 5A**, p38 MAPK inhibitor, but not JNK MAPK inhibitor, significantly inhibited IFN-β expression in cells with pEGFP-*bfr* transfection (*p* < 0.001). It is well known that the activation of p38 pathway requires phosphorylation of p38 (Wang et al., 1997). We therefore examined whether p38 is phosphorylated during Bfr expression. We transfected DF-1 cells with pEGFP-*bfr*, and examined the phosphorylation of p38 in pEGFP-*bfr* transfected cells with Western Blot using specific antibodies against p-p38, p38, and GAPDH. As a result, p38 phosphorylation was markedly enhanced in pEGFP-*bfr* transfected cells (**Figures 5B–D**). Taken together, these results clearly show that Bfr-induced phosphorylation of p38 is involved in induction of IFN-β expression. Thus the p38 MAPK pathway is essential for Bfr-induced IFN-β expression.

### Bfr Plays a Critical Role in *S. pullorum*-Induced IFN-β Expression in Cells

**FIGURE 6:**
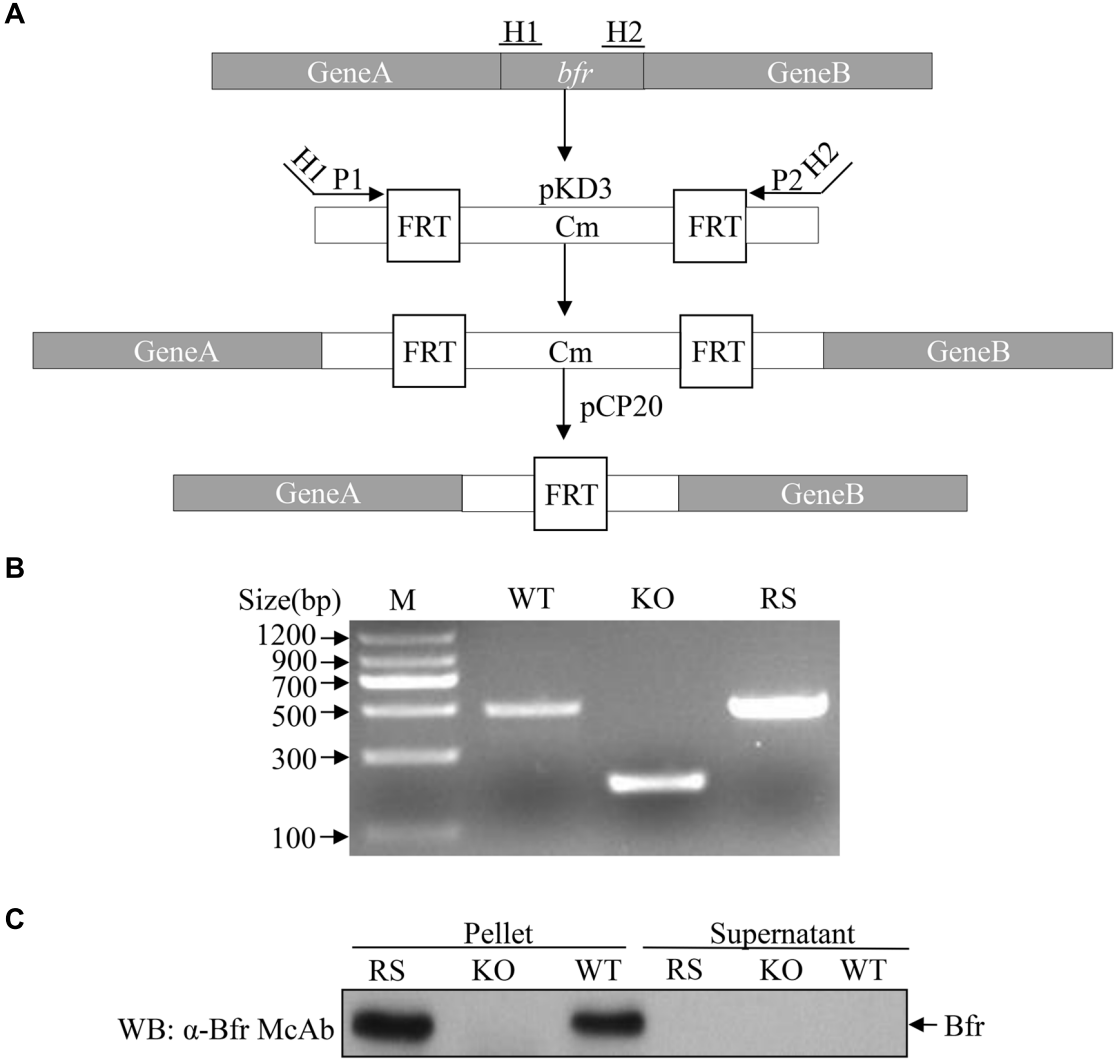
**Generation of Bfr-deficient *S. pullorum* strain by Red homologous recombination. (A)** Schematic diagrams showing the strategy for the deletion of *bfr* gene. **(B)** Identification of recombinant (KO) and complemented (RS) strains by PCR. PCR was performed to examine *bfr* gene in WT, KO, and RS *S. pullorum* strains. **(C)** Examination of Bfr expressions in WT, KO, and RS *S. pullorum* strains. Cell lysates of WT, KO, and RS *S. pullorum* were prepared, and Bfr expressions were examined with Western Blot using anti-Bfr monoclonal antibody.

The fact that Bfr induced expression of IFN-β in host cells prompted us to examine the role of Bfr in *S. pullorum-*induced IFN-β response in cells. We generated a Bfr-deficient *S. pullorum* strain using λ-Red-mediated recombination system according to a simple gene disruption strategy (**Figure 6A**) and also generated the ΔBfr-complemented strain expressing Bfr by electroporation of Bfr-deficient *S. pullorum* with pBR322-*bfr*. As shown in **Figures 6B,C**, WT and the complemented *S. pullorum* strains expressed Bfr very well. In contrast, Bfr-deficient *S. pullorum* had no detectable Bfr as examined by PCR and Western Blot. These results indicate that Bfr-deficient *S. pullorum* strain and its complemented strain expressing Bfr were successfully generated.

**FIGURE 7:**
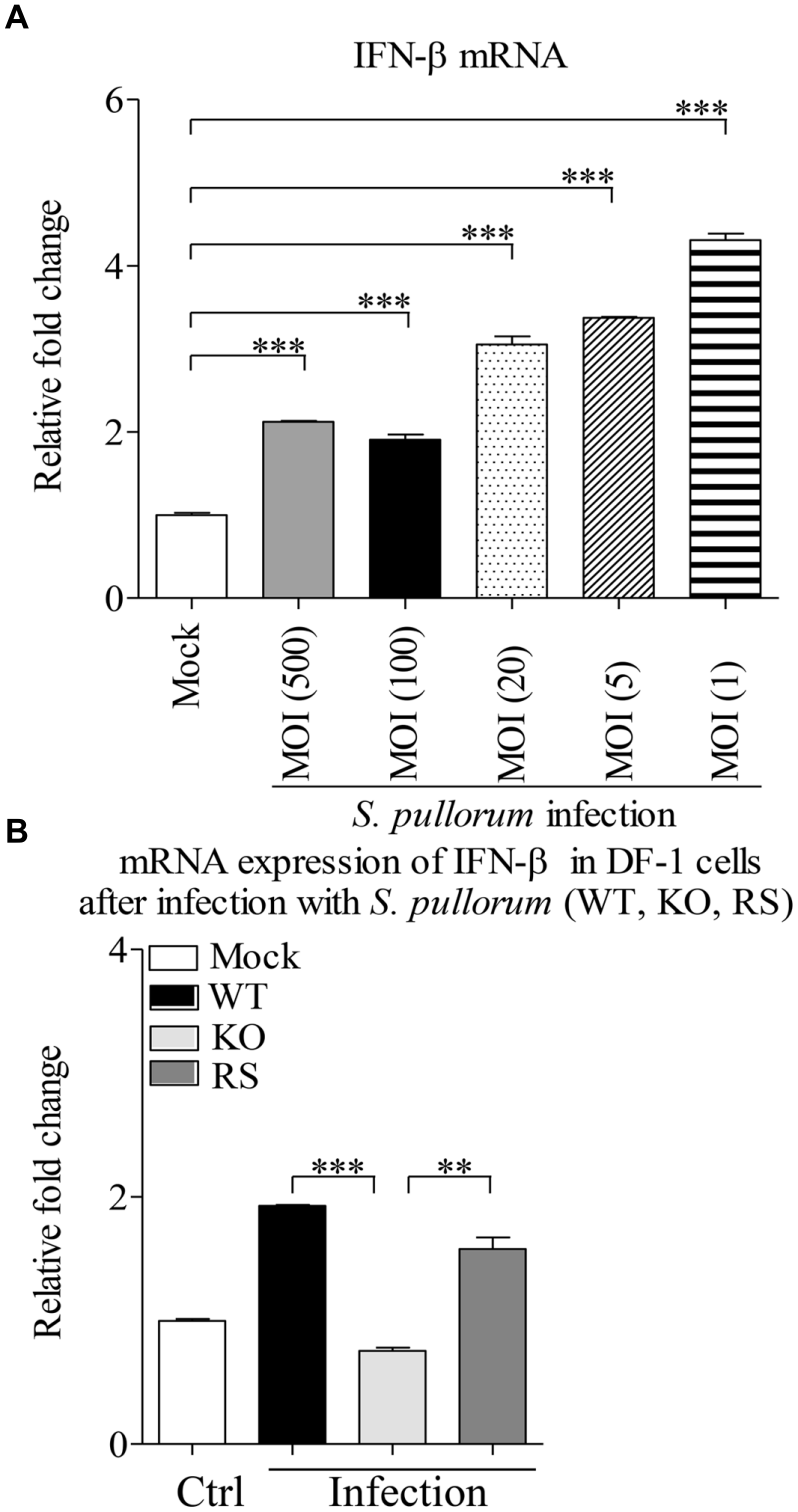
**A critical role of Bfr in *S. pullorum*-induced IFN-β expressions. (A)** DF-1 cells were infected with WT *S. pullorum* at an indicated MOI, 8 h after infection, mRNA expressions of IFN-β in cells were examined with qRT-PCR using specific primers. The expression levels of mRNA were calculated in relation to the expression level of GAPDH. **(B)** DF-1 cells were infected with WT, KO, and RS *S. pullorum* strains at an MOI of 500. Eight hours after infection, mRNA expressions of IFN-β in cells were examined with qRT-PCR using specific primers. The mRNA expression levels of IFN-β in cells were calculated in relation to the expression level of GAPDH. Results are representative of three independent experiments. Data are represented as mean ± SD, *n* = 3. ^∗∗∗^ *p* < 0.001 and ^∗∗^ *p* < 0.01.

We cultured WT, Bfr-deficient and the complemented *S. pullorum* strains in culture medium, and compared their growth at 0, 6, 12, and 24 h after culture. As a result, we did not find any difference between WT, Bfr-deficient and the complemented *S. pullorum* strains in terms of their growth (data not shown), suggesting that deficiency of Bfr in *S. pullorum* does not affect the bacterial replication. We infected DF-1 cells with WT *S. pullorum* at an MOI of 1, 5, 20, 100, or 500. Eight hour after infection, the mRNA expression of IFN-β was examined with real-time PCR assay. As shown in **Figure 7A**, infection of DF-1 cells with WT *S. pullorum* markedly induced mRNA expression of IFN-β in cells (*p* < 0.001). However, WT *S. pullorum-*induced IFN-β expression was completely abolished by deletion (knock-out) of *bfr* gene from the bacteria (**Figure 7B**), indicating that Bfr is required for *S. pullorum-*induced IFN-β expression. Thus, Bfr plays a critical role in *S. pullorum*-induced innate response in host cells.

## Discussion

*Salmonella pullorum* is a worldwide distributed poultry pathogen of considerable economic importance to the poultry industry, particularly to that of developing countries. An increasing number of *S. pullorum* strains were isolated in China, and many of these bacteria showed antimicrobial resistance (Pan et al., 2009). This pathogen causes high mortality in young chickens and persistent infection in adult chickens with clinical signs of decreased egg production and diarrhea. Although the humoral immune response against *S. pullorum* cannot clear the pathogens once the bacteria intrude host cells, the specific antibodies still play an important role in mediating phagocytosis of extracellular bacteria by phagocytes. In particular, the examination of anti-*S. pullorum* antibodies is of clinically diagnostic importance. Thus hemagglutination assay with the whole blood of chickens is generally performed to screen for the *S. pullorum*-infected chickens in flocks. Immunoprecipitation of whole bacterial cell lysate by antibodies against *S. pullorum* (Pull-down assay) is an efficient method that allows us to identify major antigens of the microorganism that elicit humoral immune response. The identified antigen would help to develop new diagnostic methods or vaccine in control of the disease (Britton et al., 1988; Sexton et al., 1991). High titers of anti-*Salmonella* IgY were produced by birds infected with *S. pullorum* after 5 weeks (Wigley et al., 2001). However, little information is available regarding the major antigen of *S. pullorum.* In this study, a major antigen Bfr that elicited antibody response was identified with a pull-down assay and Mass spectrometric method. Our results demonstrate that recombinant Bfr can be specifically recognized by pullorum-positive serum. Thus, Bfr-induced antibodies probably play an important role in host response against *S. pullorum* infection.

Bfr, a 17-kDa protein that was previously identified as an outer membrane protein of *S. hadar*, is composed of 24 identical subunits along with the usual ferroxidase sites that have 12 binding sites for heme iron (Wong et al., 2009; Snoussi et al., 2012). Bfr is also the major Fe storage protein in *Salmonella* (Velayudhan et al., 2007). We could observe the color of Fe^3+^ from the purified Bfr-his recombinant protein in our experiments. Inactivation of Bfr induces intracellular free Fe concentration and exhibits increased susceptibility to oxidative stress (Velayudhan et al., 2007). It was found that the transcription factor SsrB controls resistance to reactive oxygen species through Bfr in *Salmonella* (Brown et al., 2014). This information suggests that Bfr is involved in regulation of iron homeostasis and protects against hydrogen peroxide toxicity in bacteria. H_2_O_2_ can react with Fe^2+^ ions via Fenton and Haber-Weiss reactions, producing ROS (reactive oxygen species) such as hydroxyl radical or superoxide, which are capable of damaging most cellular components, such as nucleic acids, protein or membrane lipids (Timoteo et al., 2012). And Bfr binds to DNA and reduces the damage of ROS by the rapid uptake and oxidation of free Fe^2+^, using H_2_O_2_ as the oxidant (Timoteo et al., 2012). Our results demonstrate that recombinant Bfr can mediate the rapid uptake and oxidation of free Fe^2+^, using H_2_O_2_ as the oxidant. Interestingly, we also found that Bfr could induce self-activation in a yeast two-hybrid screening and apoptosis in DF-1 cells in this study (data not shown). This information suggests that Bfr might be a muti-functional protein.

Bacterioferritin is an important factor in bacteria, but few reports are available regarding the cell response to Bfr. Currently it is known that Bfr is a T-cells antigen that induces a strong IFN-γ production and the proliferation of lymphocytes (Denoel et al., 1997; Al-Mariri et al., 2002; Lee et al., 2006). In addition, Bfr induced humoral immune response in mice immunized with DNA vaccine encoding the Bfr or recombinant Bfr proteins (Al-Mariri et al., 2001a,b). The patient sera of *M. leprae* could react with Bfr, and *M. paratuberculosi* antigen D was identified as Bfr (Brooks et al., 1991; Spencer et al., 2011). These findings suggest that Bfr plays an important role in acquired immunity. However, little is known about the role of Bfr in the innate immune response. Our data show that Bfr not only acts as a potent antigen inducing humoral immune response but also as an inducer for innate immune responses (inducing IFN-β expression), indicating that Bfr is not merely a protein for iron storage and detoxification (Bou-Abdallah et al., 2002). Furthermore, we found that the amino acids 1–50 of Bfr were responsible for induction of IFN-β expression. However, Bfr did not affect the expression of IFN-α in cells. It seems that the role of Bfr is specific, only for induction of IFN-β expression.

Recognition of bacterial products by host surveillance system results in transcription of the *ifnb* gene, and the activation of cytosol-specific signaling is associated with phosphorylation of the p38 mitogen-activated protein (MAP) kinase (O’Riordan et al., 2002). In this study, when DF-1 cells were treated with p38 MAP Kinase inhibitor, Bfr-induced IFN-β expression was markedly inhibited, indicating that Bfr might activate cytosol-specific signaling. In contrast, JNK MAP Kinase inhibitor had no effects on Bfr-induced IFN-β response. These results suggest that Bfr induces IFN-β expression via the p38 signal transduction pathway. As p38 MAP kinases are major players during inflammatory responses, they can be activated by environmental and cellular stresses including pathogens, heat shock, growth factors, osmotic shock, ultraviolet irradiation and cytokines (Yang et al., 2014). p38 kinases have two domains: a 135 amino acid N-terminal domain and a 225 amino acid C-terminal domain. The phosphorylation lip of p38 consists of 13 residues, Leu-171-Val-183, and the protein is activated by phosphorylation of a signal threonine (Thr-180) and a single tyrosine residue (Tyr-182) in the lip (Wang et al., 1997). Furthermore, we found that p38 phosphorylation was induced by Bfr, indicating that p38 signal transduction pathway is essential for IFN-β expression in cells.

DF-1, an immortal chicken embryo fibroblast cell line, is commonly used for the research of *Salmonella* (Li et al., 2006; Szmolka et al., 2015) and type I interferon (Li et al., 2013). Our data show that *S. pullorum* infection significantly induces activation of the IFN-β promoter in DF-1 cells, supporting the previous publication by Hess et al. (1989). In contrast, *S. pullorum*-induced IFN-β expression was completely abolished by deficiency of Bfr in the bacteria, indicating that Bfr is required for *S. pullorum*-induced IFN-β expression in cells.

IFN-β is a key cytokine in the innate immune response, mediating expression of hundreds of IFN-stimulated genes (ISGs) that are responsible for the establishment of an antimicrobial state in the infected tissue (Schmolke et al., 2014). It was reported that IFN-β potently represses *S. typhimurum-*dependent induction of IL-1 family cytokines and neutrophil chemokines and IFN-β^-/-^ mice exhibit greater resistance to oral *S. typhimurum* infection and a slower spread of *S. typhimurum* to distal sterile sites (Perkins et al., 2015). *S. typhimurum* induces the production of IFN-β, which drives necroptosis of macrophages and allows *Salmonella* to evade the immune response that is detrimental to the survival of mice (Robinson et al., 2012). Since Bfr induced IFN-β expression through the p38 MAP Kinase signaling pathway in cells, several important questions need to be addressed. For example, what is the host protein directly targeted by Bfr? What is the role of IFN-β induced by Bfr in host cell response to *Salmonella* infection? Elucidation of these questions will further our understandings of the mechanisms underlying pathogenesis of *Salmonella* infection.

## Conclusion

Our results demonstrate that Bfr is a major antigen of *S. pullorum.* Our data also show that Bfr induced IFN-β expression via its amino acids 1–50 portion. Furthermore, we found that the p38 MAPK signaling pathway was essential for Bfr-induced IFN-β expression. Importantly, *S. pullorum* induced-IFN-β was totally abolished by deficiency of Bfr in the bacteria, indicating that Bfr plays a critical role in *S. pullorum* induced-IFN-β expression in DF-1 cells. Our findings provide new insights into the molecular mechanisms of the host response to *S. pullorum* infection.

## Author Contributions

SZ and ZX conceived and designed the experiments; ZX performed the experiments; SZ and ZX analyzed the data; SZ, YQ, YW, XL and HC contributed reagents/materials/analysis tools; SZ and ZX wrote the paper.

## Conflict of Interest Statement

The authors declare that the research was conducted in the absence of any commercial or financial relationships that could be construed as a potential conflict of interest.
